# Weight-Loss Interventions for Hispanic Populations: The Role of Culture

**DOI:** 10.1155/2013/542736

**Published:** 2013-02-26

**Authors:** Nangel M. Lindberg, Victor J. Stevens, Ruben O. Halperin

**Affiliations:** ^1^Kaiser Permanente Center for Health Research, 3800 N. Interstate Avenue Portland, OR 97227-1110, USA; ^2^Providence Medical Center, 5050 N.E. Hoyte St. Suite 540, Portland, OR 97213, USA

## Abstract

In the United States, ethnic minorities are overrepresented among the overweight and obese population, with Hispanic individuals being among the groups most at risk for obesity and obesity-related disease and disability. Most weight-loss interventions designed for the general population have been less successful with individuals from ethnic minorities and there is a pressing need to develop more effective interventions for these groups. This paper examines the importance of culture in the development of “culturally competent” weight-loss interventions for ethnic minority populations, and discusses specific culturally mediated factors that should be considered in the design and implementation of treatment interventions. While specifically focusing on Hispanic populations, we also address issues of relevance to other multiethnic societies.

## 1. Introduction

The harmful consequences of obesity have been well documented: obesity increases overall mortality [1–3], decreases life expectancy [4], and is an important risk factor for diabetes and cardiovascular disease, and many other conditions [5–9]. Obesity and its consequences disproportionately affect ethnic minority communities [10–13], with individuals of Hispanic origin, particularly Mexican-Americans, among the groups at highest risk [5, 14–17]. 

In the United States, newly arrived immigrants from Spanish-speaking countries are generally healthier than the U.S.-born Hispanic population, but as their duration of residency increases, so does the prevalence of poor health behaviors [18, 19], including the adoption of high-fat, low-fiber diets [20]. As a consequence, there is a linear association between obesity and length of residence in the United States for Mexican immigrants; those with 15 years or longer of residence have a fourfold greater risk of obesity compared to those who have been here for less than 5 years [20].

## 2. Diet, Culture, and Weight Loss

Culture is intimately tied to diet. It has been suggested that there is perhaps no better way to understand a culture, its values, preoccupations, and fears than by examining its attitudes toward food [21]. Food not only provides daily sustenance but also provides a core element which bonds families and communities and provides a common element to mark rites of passage and celebrations. Selection of ingredients, how foods are prepared, the timing and context of meals, size of portions, notions of healthful versus unhealthy foods, and what is considered a “meal” and what is considered a “snack,” are all integral parts of cultural patterns. Dietary patterns separate individuals and groups from one another and are an important component of cultural and national identity [22].

Despite the close association between diet and culture, and the fact that individuals of Hispanic origin have a particularly high prevalence of overweight and obesity, few published reports are available on weight-loss interventions specifically targeting Hispanic populations. A comprehensive review of the literature [23] showed that, overall, weight-loss interventions designed for the general population are generally less successful for Hispanics. It is difficult to draw conclusions from this general finding given that, to date, most weight-loss studies with Hispanic participants have provided little or no information on how Hispanic ethnicity was determined and have frequently failed to examine or report key variables such participants' countries of origin, acculturation, level of education, urban/rural origin, or the confounding effects of SES and ethnicity [23].

Most of the interventions described as “culturally adjusted” have not provided information regarding their specific cultural adjustments, although it often appears that materials used in general interventions had been translated into Spanish, or that the interventions were conducted by bilingual or “Hispanic” facilitators [23]. Beyond delivery of the intervention in Spanish, the two most salient cultural adaptations appear to be employing materials appropriate for low literacy levels and offering family-based interventions with the implications that low literacy levels are inherent in Hispanic cultures and that familism is a uniquely and universally held Hispanic value, a notion that has been challenged in the literature [24, 25]. Not surprisingly reviews have found that most “culturally adjusted” interventions targeting Hispanics appear minimally guided by cultural frameworks [26].

## 3. Culture and Weight-Loss Interventions

Many elements central to weight-loss interventions (e.g., food choices, behavioral strategies, portion control) are closely tied to culture and social context. Interventions targeting general populations are not usually described as “culturally adjusted,” but of course they reflect both the cultural values of the intervention developers and the previous experience of those developers with the target population. A particularly relevant example for this discussion is the area of food measurement. Most weight-loss interventions conducted in the United States rely heavily on accurate food measurement as the center piece of portion control strategy, and for most white, non-Hispanic participants, food measurement is not a new skill. However, precise food measurement is not entirely congruent with traditional Mexican culture, where food preparation rarely involves the use of standard measurements. Family recipes and traditional Mexican cookbooks rarely include precise measurements of ingredients; instead, food preparation is often done “to taste,” and amounts are “guesstimated” (“*calculado a ojo*”) [27]. The Spanish language, in fact, lacks specific terms for “tablespoon” or “teaspoon”; instead, spoons are defined according to use (“*cuchara sopera,*” referring to a soup spoon, “*cuchara de postre*,” referring to a dessert spoon), and the differing sizes of spoons are denoted by attaching a diminutive suffix to the basic word “spoon.” Thus, “*cuchara*” (spoon) becomes “*cucharita*” (little spoon), a term used to describe *any* spoon of relatively small dimensions, including—but not exclusively—the English measurement unit known as a “teaspoon.” 

Weight-loss interventions targeting the general, English speaking population commonly caution against eating as a reaction to stress, describing the practice as a “maladaptive coping strategy.” However, among many Hispanic populations, eating is a *culturally sanctioned* form of stress-reduction, and eating bread or sugar is, in fact, a common home remedy, specifically prescribed by both practitioners of traditional Mexican medicine as well as conventional Mexican physicians to prevent ill-effects of trauma [28, 29]. This centuries-old practice is illustrated by the traditional Spanish saying that “grief is lessened by bread” (“*las penas con pan son menas*”). 

## 4. Cultural Factors Affecting Weight Loss 

To develop effective weight-loss interventions for different ethnic groups, it is essential to first identify and understand culturally relevant factors likely to affect the outcomes of these interventions (e.g., body image, personal disclosure, characteristics of intervention settings, etc.) and allow that understanding to guide the development of interventions. For example, individuals' views of desirable weight and body shape are important culturally mediated factors affecting weight loss; in the midst of public health concerns regarding the obesity epidemic among Hispanic children [30–32], several studies have found that Hispanic women choose a plump figure as ideal for their children and associate overweight children with health and vitality, and thinness with poor health and vulnerability to illness [32–34]. These views are likely to impact Hispanic women's interest in, and adherence to, weight-loss intervention programs targeting their children. In another example of the need to focus on cultural context, studies have found that immigrants from Spanish-speaking countries gradually stop adhering to their traditional—and often more healthful—dietary practices [35] not only because of often-cited long work schedules or lack of access to fresh fruits and vegetables, but also because they have gained access to certain foods (such as meat or desserts) which were previously unaffordable and because, for some immigrants, “junk food is cool” [36, 37]. Effective weight-loss interventions for Hispanic immigrants must understand the meaning of the so-called “American food” and the role it plays in the acculturative process. Our efforts aimed at promoting healthful diets among Hispanic immigrants are likely to be of limited effectiveness if we disregard the fact that, for many, the consumption of processed and fast foods remains a symbol of modernization or their assimilation into American society.

## 5. Development of Effective Culturally Competent Interventions

Food choices are affected by culturally mediated health beliefs and by popular food classifications. Some Hispanic groups uniquely classify food based on factors constructed from sensory data and based on intrinsic qualities of the foods and their balance in the human body, such as “hot-cold,” and “male-female” [38]. In many Hispanic cultures, factors such as the person's gender or reproductive status, or whether the food is classified as “safe” (foods that don't make one sick), “tonic” (thought to be good for one's health), or “dangerous” (thought to be potentially unhealthy, depending on the time of day when they are consumed) often dictate the consumption or avoidance of foods. Examples of this are found in the traditional Spanish sayings “*la naranja en la mañana es oro, en la tarde plata, y en la noche, mata”* (oranges are gold in the morning, silver in the afternoon, and deadly at night), or “*ajo, cebolla, y limón y déjate de inyección”* (garlic, onion, and lemon, and forget all medicines), or in the beliefs that, if consumed at certain times, or in certain combinations, some foods will stick to a child's stomach and cause *empacho*, or that failure to satisfy *antojos* (food cravings) by pregnant women may cause harm to the unborn baby [28].

## 6. Heterogeneity of the Hispanic Population

Much of the information about the health status of ethnic minorities in the United States has treated Hispanics as a single, monolithic population, without exploring the variation by ethnic ancestry [39]. However, when these differences are examined, Hispanics emerge as a highly heterogeneous group, with differences in health-care service utilization and medical outcomes associated with their country of origin, preferred language, and length of time living in the United States [39]. To develop effective treatment interventions for this population it is imperative to acknowledge the enormous cultural, linguistic, acculturative, economic, and educational diversity of the Hispanic population, and focusing on “Hispanic foods,” is far from sufficient to make an intervention “culturally appropriate.” The examples abound: most dietary change interventions targeting Hispanics focus on corn as a staple food, but while corn is the chief staple food in Mexican culture, it plays a negligible role in many Caribbean and South American cultures, whose nutritional cornerstones are cassava, rice, and plantains [40, 41]. Culturally tailored interventions often ignore the heterogeneity of the Spanish language. For example, the inclusion of “*choclo*” (a uniquely Argentinian term for corn) in the Spanish version of the Diabetes Prevention Program (DPP) [42] food guide was most likely puzzling to participants of Mexican origin, for whom “*choclo*” is a thick-soled shoe, and corn is called “*elote*” or “*maiz*.”

## 7. Problems with Dietary Assessment Tools

Food frequency questionnaires (FFQs) are an important part of weight loss research. Because these tools depend on a predetermined list of food items, they reflect the dietary habits of the population for which they were developed [43]. While efforts have been made to adapt existing instruments to assess the dietary intake of Hispanic populations, some of the assumptions made for specific food items are far off the mark for different cultural groups. For example, the widely used Spanish version of the Block questionnaire [44] groups under a *single line item* four largely dissimilar food items (“tacos, burritos, enchiladas, tamales”) whose only common element is their popular classification as “Mexican foods.” This would be akin to an FFQ that combines under a single line item “French fires, hamburger, meatloaf, pancake, macaroni and cheese” based on the fact that they are all considered “American foods.” To assess consumption of corn tortillas, this culturally adapted version of the Block FFQ makes “four tortillas in one day” the *highest* possible choice ignoring the fact that, on the average, Mexican adults report a mean daily intake between 300 and 400 g of corn tortillas, which translates into approximately 8 to 18 tortillas per day [45, 46]. Highly Anglocentric weight loss interventions that make dietary recommendations that are so far removed from the actual practice of the target population and that ignore the cultural significance of key food items, are likely to be met with skepticism and may result in poor participant retention rates. 

Some advances have been made in the development and validation of food frequency questionnaires (FFQs) for different ethnic populations [47], including the addition of regionally relevant food items, modification of frequencies of intake, and updating of nutrient data bases [43, 48, 49]. However, our experience is that the use of FFQs among Hispanic populations is likely to result in biased estimates of intake. This is due to the large diversity of regional foods that play a central role in the diet of Hispanic subpopulations and the difficulty of any one instrument to capture the majority of key food items for each regional diet, along with accurate frequencies of intake and nutrient data. For example, the inclusion of “tacos” as an item in a culturally adapted FFQ ignores the fact that in Mexican culture a “taco” simply denotes a rolled-up tortilla onto which some food—any food—has been added. Thus, a warm tortilla sprinkled with salt, becomes a “salt taco,” and a tortilla filled with a rich chicken dish, rolled up, deep fried, and garnished with lettuce, avocado, sour cream, and cheese is also a taco—a “chicken taco.” The obvious differences in caloric and macronutrient content of each of these two “tacos” are not captured by the FFQ format. The equivalent of this problem would be an FFQ where there is a single and unspecified item “sandwich” which does not distinguish the caloric and macronutrient differences between a 75-kcal single-slice peanut butter sandwich and a 500-kcal Philly cheese steak sandwich.

## 8. Useful Approaches in the Development of Culturally Appropriate Interventions

It has long been recognized that treatments are more effective when they are personalized to the needs and context of the individuals. A great deal of progress has been made in the methodology of cultural adaptations of interventions [50, 51]. In the field of weight-loss interventions, the first steps in developing effective culturally tailored interventions must start with identifying the target population, learning about their views on weight and body shape, and understanding the preferences, customs, and beliefs that rule their consumption of food. This approach was used by Karanja and colleagues in designing a weight-loss intervention for African American women [52]. Rather than starting with preconceived notions of what elements would be effective, or “transposing” materials to fit assumptions about what “would work” with an African American population, the study began with focus groups, which provided information about African American women's beliefs and needs regarding weight-loss interventions and guided the development of a culturally tailored intervention that incorporated elements suggested by the participants. Karanja's project was associated with greater weight losses and higher participant retention rates than those that had been observed in other weight-loss programs targeting African American women [52].

Another useful approach may be found in the PEN-3 health education model [53], which incorporates frameworks in health belief and health promotion, while drawing on theory and application in cultural studies, establishing culture as the core of health promotion and prevention programs. The model consists of three dimensions: identification of target population, exploration of the target groups' perception of health information and the factors that enable their health-related behaviors, and determining the cultural appropriateness of health behaviors. This model, which has elements in common with Karanja's approach, has been used to assess cultural eating patterns among African Americans [54] and to identify segments of the population that should be targeted for nutrition education programs [55]. Much could be gained from exploring its use among Hispanic individuals. 

## 9. The Problematic Nature of the Term “Hispanic”

Given the complexity of ethnic identity and the intricate and dynamic interplay between culture of origin and acculturation, it is virtually impossible to talk about “Hispanics” with any precision. Describing a group as Hispanic is about as precise and useful as defining a group as “American.” While “American” may be a useful geopolitical term, it provides virtually no information about the practices, beliefs, habits, or genetic information of such a large heterogeneous group. When developing health-promotion interventions targeting what are often heterogeneous Hispanic populations, it is essential to acknowledge the complexity of ethnic identity, the diversity of Hispanic cultural groups, and the dynamic interaction between culture of origin and acculturation and to align recommendations with the specific health beliefs, dietary patterns, behavioral patterns, social networks, and immigration-related experiences of the target population. For example, culturally tailored interventions specifically targeting migrant farm workers from Central America are likely to address dietary habits, physical activity practices, and social and economic barriers that are unlikely to be of any relevance to fifth generation middle-class Texans of Mexican origin or college-educated Argentine immigrants.

## 10. Proposed Guidelines for Researchers

To address the need to adequately adapt weight-loss interventions for a wide diversity of Hispanic populations, we propose some preliminary guidelines to follow in the development of weight-management interventions.

- The ill-defined category of “Hispanic/Latino” should be avoided as a grouping variable. Instead, researchers should identify what specific cultural characteristics of a particular group (such as their specific regional origin, their length of residence in the United States) justify clustering participants into one group.
- Given that factors such as education level, income, country of origin, length of residence (or generation) in the United States are strongly associated with health outcomes, these variables should be closely examined and, if appropriate, considered as alternative explanatory variables. Differences observed between racial or ethnic groups should not automatically be attributed to ethnicity, race, or culture.
- Researchers should avoid the automatic assumption of “Hispanic values” (e.g., familism, specific religious identification, food preferences, fatalism, or so-called “confianza”). Such factors must be explored and ascertained, rather than automatically assumed. We propose the use of focus groups or serial qualitative interviews with individuals from the target population as a way to explore their socio-cultural context, belief systems, and common practices. This in turn can inform and guide intervention development and cultural adaptation processes. This approach has been used successfully in the development of intervention tools for specific patient populations [56, 57], including weight-loss interventions with ethnic minority populations [27, 52, 58]. Similarly, researchers should avoid the automatic assumption that certain values or practices (e.g., standard food measurement, dietary concepts, health beliefs, or food classifications) are universal. Just like interventions targeting teens, pregnant women, or the elderly generally differ in format, content, frequency, and materials in order to address the specific needs and preferences of each of those groups, the same careful tailoring must be applied to establish the specific preferences, needs, and skills of individuals of diverse cultural backgrounds.
- Interventions should include multiple staff members who are thoroughly familiar not only with the participants' preferred language, but also with their culture of origin. Culturally competent individuals can not only navigate the subtle nuances of Spanish language (and thus avoid the “*choclo*” versus “*elote*” confusion) but can also make dietary recommendations that “make sense” to, and are thus more likely to be followed by, the target population.
- We believe that dietary food recalls conducted by culturally competent interviewers are the best tool for dietary assessment in Hispanic populations.
- While cultural adaptations may make it possible to provide effective interventions for individuals of different ethnic backgrounds, there is a paucity of data on the relative importance of different elements of cultural adaptation. The identification of the key elements in culturally adapted interventions that are more closely associated with successful outcomes remains an important objective.
- Researchers must remember that diet is much more than the individual nutrients that feed us. It is also a form of communication and a vehicle through which values are passed on from one generation to the next.
